# Generation, annotation, analysis and database integration of 16,500 white spruce EST clusters

**DOI:** 10.1186/1471-2164-6-144

**Published:** 2005-10-19

**Authors:** Nathalie Pavy, Charles Paule, Lee Parsons, John A Crow, Marie-Josee Morency, Janice Cooke, James E Johnson, Etienne Noumen, Carine Guillet-Claude, Yaron Butterfield, Sarah Barber, George Yang, Jerry Liu, Jeff Stott, Robert Kirkpatrick, Asim Siddiqui, Robert Holt, Marco Marra, Armand Seguin, Ernest Retzel, Jean Bousquet, John MacKay

**Affiliations:** 1 ARBOREA and Canada Research Chair in Forest Genomics, Pavillon Charles-Eugène-Marchand, Université Laval, Ste.Foy, Québec G1K 7P4, Canada; 2 Center for Computational Genomics and Bioinformatics, University of Minnesota, 420 Delaware St. S.E., MMC 43, Minneapolis, MN 55455, USA; 3 Laurentian Forestry Center (Canadian Forestry Service), Natural Resources Canada, 1055 rue du PEPS, Québec, Québec, G1V 4C7, Canada; 4 Genome Sciences Center, BC Cancer Agency, 675 West 10 th Avenue, Vancouver, BC, V5Z 1L3, Canada; 5 Department of Biological Sciences, University of Alberta, Edmonton, Alberta, T6G 2E9, Canada

## Abstract

**Background:**

The sequencing and analysis of ESTs is for now the only practical approach for large-scale gene discovery and annotation in conifers because their very large genomes are unlikely to be sequenced in the near future. Our objective was to produce extensive collections of ESTs and cDNA clones to support manufacture of cDNA microarrays and gene discovery in white spruce (*Picea glauca *[Moench] Voss).

**Results:**

We produced 16 cDNA libraries from different tissues and a variety of treatments, and partially sequenced 50,000 cDNA clones. High quality 3' and 5' reads were assembled into 16,578 consensus sequences, 45% of which represented full length inserts. Consensus sequences derived from 5' and 3' reads of the same cDNA clone were linked to define 14,471 transcripts. A large proportion (84%) of the spruce sequences matched a pine sequence, but only 68% of the spruce transcripts had homologs in *Arabidopsis *or rice. Nearly all the sequences that matched the *Populus trichocarpa *genome (the only sequenced tree genome) also matched rice or *Arabidopsis *genomes. We used several sequence similarity search approaches for assignment of putative functions, including *blast *searches against general and specialized databases (transcription factors, cell wall related proteins), Gene Ontology term assignation and Hidden Markov Model searches against PFAM protein families and domains. In total, 70% of the spruce transcripts displayed matches to proteins of known or unknown function in the Uniref100 database (*blastx *e-value < 1e-10). We identified multigenic families that appeared larger in spruce than in the *Arabidopsis *or rice genomes. Detailed analysis of translationally controlled tumour proteins and S-adenosylmethionine synthetase families confirmed a twofold size difference. Sequences and annotations were organized in a dedicated database, SpruceDB. Several search tools were developed to mine the data either based on their occurrence in the cDNA libraries or on functional annotations.

**Conclusion:**

This report illustrates specific approaches for large-scale gene discovery and annotation in an organism that is very distantly related to any of the fully sequenced genomes. The ArboreaSet sequences and cDNA clones represent a valuable resource for investigations ranging from plant comparative genomics to applied conifer genetics.

## Background

Genomics projects have been initiated in several pine and spruce species to identify genes involved in traits of economic interest and of ecological significance in conifers. It is unlikely, however, that conifer genomes will be completely sequenced in the near future because of their shear size [1]. For example, estimates of the haploid DNA content of *Pinus taeda *ranged from 11 pg [2] to 23.2 pg [3] and that of *Picea glauca *ranged between 4.5 pg [4] to 20.2 pg [PGI5.0; [5]]. With around 10–20,000 Mb [6], conifer genomes are more than 100 times larger than that of *Arabidopsis *and three times larger than the human genome. Such a large genome suggests that strategies that aim at characterizing the coding component of the genome will be more cost efficient for the recovery of information, in the short term.

The large-scale sequencing and analysis of ESTs remain a fundamental part of genomics research to enable gene discovery and annotation in most forest tree species, but especially in conifers. Several EST sequencing projects have been initiated in pines; 191,229 ESTs from several species were assembled to produce 35,053 consensus sequences in the Pinus Gene Index [7]. A large majority of conifer sequences were shown to have sequence similarity to Angiosperm genes or genome sequences like *Arabidopsis*, however the identification of homologous sequences depends largely on the length of sequences available to conduct similarity searches [8,9]. In loblolly pine, for example, the majority of contigged sequences which had no sequence similarity to other genomes were very short and more than 90% of sequences above 1 kb in length gave strong matches to *Arabidopsis *[8]. Therefore, effective annotation of conifer coding sequences through comparative approaches is best achieved with complete information, which may be obtained by combining 3' and 5' sequences or by full length sequencing strategies. A recent investigation of the *knox *gene family in conifers showed that gene evolution and conifer protein family structure may diverge quite significantly from those of Angiosperm genomes [10]. It is unknown how widespread this phenomenon may be; however, the finding suggests that although conserved protein motifs may be unambiguously identified, the biological role of genes belonging to conifer protein families may not be readily inferred from their Angiosperm homologs. These data would support the argument in favour of thorough cDNA sequencing projects in conifers because they are distantly related to model Angiosperms like *Arabidopsis*, in order to fully characterize protein families.

Many conifer EST sequencing projects have focused on wood formation and secondary xylem in pines (e.g. due to the ecological significance of the genus and the economic importance of wood [8,11]). More recently, programs have emerged that involve other species including Douglas-fir [12] and spruce [13], and address other important aspects of tree physiology like the response to abiotic stresses or biotic stresses [12,14]. Macroarrays and microarrays ranging in scope from a few hundred to a few thousand genes have been developed to help identify genes involved in wood formation and to characterize their putative roles in determining wood quality (e.g. in maritime pine [15], and in loblolly pine [16]). The relatively high level of sequence similarity between genera within the Pinaceae family has lead to the use of loblolly pine arrays for expression profiling experiments in scots pine, norway spruce [17] and white spruce [18]. Transcript profiling has also been integrated into investigations of xylem differentiation in poplar [19], different questions related to wood formation have also been investigated by transcript profiling in Angiosperm trees, including heartwood of black locust trees [20], tissue differentiation in poplar [19] and tension wood formation in *Eucalyptus *[21].

Spruce is the most widely used genus for forest tree plantations in Canada, with hundreds of million seedlings planted each year [22]. It is also widely divergent from pine [23,24]. Genetic improvement of spruce species, mainly white and black spruces, has been ongoing in Canada since the 1950s and extensive information has been accumulated on the genetic control of commercially important traits. Genome mapping of spruces is underway to enable molecular breeding applications (e.g. [25]). Association mapping approaches have been proposed as most promising to identify genes underlying phenotypic variation in quantitative traits, and thus, to support the development of molecular breeding strategies in conifers [26]. Large-scale EST sequencing and analysis are expected to enable association studies and gene mapping research as they are prerequisite steps to identifying SNPs to use in high throughput genotyping assays.

The objective of this study was to produce extensive collections of EST sequences and cDNA clones to support manufacture of cDNA microarrays and gene discovery efforts in white spruce (*Picea glauca *[Moench] Voss). This collection of ESTs constitutes an important new resource for the genomics of white spruce and related species. In this paper, we report the sequence analysis of around 71,000 sequence reads obtained through 3' and 5' sequencing of cDNAs. Comparative analyses were conducted to assign a functional annotation based upon similarities. Spruce contigs were also correlated with terms derived from the Gene Ontology [27], and similarity searches were conducted against specialized databases to identify putative transcription factors, cell wall related proteins and protein domains available in PFAM. To mine this new sequence resource, a database called SpruceDB has been developed at the Center of Computational Genomics and Bioinformatics (CCGB, University of Minnesota) [28], which supports multiple queries on the occurrence of the ESTs in the libraries and on the functional annotations.

## Results and discusion

### Library development and resulting sequences

#### Tissue sampling and EST sequencing strategies

The cDNA libraries were developed with the goal of augmenting the representation of conifer transcripts available in public databases, and to support experimental goals related to vascular development. We sequenced ESTs from 16 non-normalized cDNA libraries, synthesized from diverse spruce organs and tissues, and representing various stages of development from immature embryos to 30 year-old trees in diverse growth conditions (Table 1) [see also Additional file 1].

**Table 1:** Sequencing and quality parameters of white spruce cDNA libraries. Quality reads had a *Phred *score above 20 over at least 100 bp after vector trimming.

Libraries, treatments and tissues	Number of reads		Library quality		Sequence quality		
	3'	5'	% Empty	% >1.6 Kb	Nb of quality reads	% Quality reads	Average length of quality reads (nt)

Male strobili development sequence	1,536	1,536	4	19	2,589	84	527
Female cones development sequence	1,536	1,536	15	9	2,324	76	500
Vegetative buds development sequence	1,536	0	5	15	1,062	69	560
Secondary xylem – mature trees	4,608	4,608	10	27	7,735	84	600
Cambium, phloem – mature trees	4,608	3,072	2	8	6,705	87	635
Secondary xylem – girdled seedlings	3,072	0	9	24	1,053	69	556
Cambium to bark – girdled seedlings	1,536	1,536	NA	NA	937	31	577
Elongating root tips – saplings	1,536	1,536	6	19	1,053	69	395
Primary, secondary shoots-N treatments	3,072	1,536	16	50	3,031	66	736
Immature somatic embryos	3,072	0	4	44	2,220	72	692
Clean roots systems – N treatments	1,536	0	7	37	858	56	659
Clean roots systems – P treatments	3,072	1,536	15	19	3,776	82	705
Clean roots systems – Diurnal cycle	6,144	4,608	16	33	8,601	80	757
Root secondary xylem – mature trees	3,072	0	7	8	1,532	50	598
Annual flush shoots diurnal cycle – trees	4,608	3,072	11	10	5,164	67	658
Needles – N fertilization treatments	1,536	0	15	20	461	30	686
Total	46,848	24,576			49,101		

We sequenced close to 50,000 cDNA clones, sampling between 1,536 and 6,144 clones from each cDNA library. The library quality assessment data, the number of sequencing reactions, and the number of high quality reads for each library are presented in Table 1. All clones were sequenced from the 3' end ; in addition, 5' sequencing was carried on many clones from the libraries of highest quality or most relevant to our research goals. In total, 71,424 reads were obtained and processed to remove vector and sequences of low quality (*Phred *score below 20). We thus retained 49,101 quality reads (QR) comprised of at least 100 contiguous nucleotides with a *Phred *score above 20 (Table 1). Among the quality reads 33.5% were from secondary vascular tissues, 32.2% were from roots, 16.7% from young shoots (all tissues), and the remaining 17.6% were from various organs including male strobili, female cones, buds, somatic embryos, and needles (Table 1).

#### EST assembly into contigs

**Figure 1:**
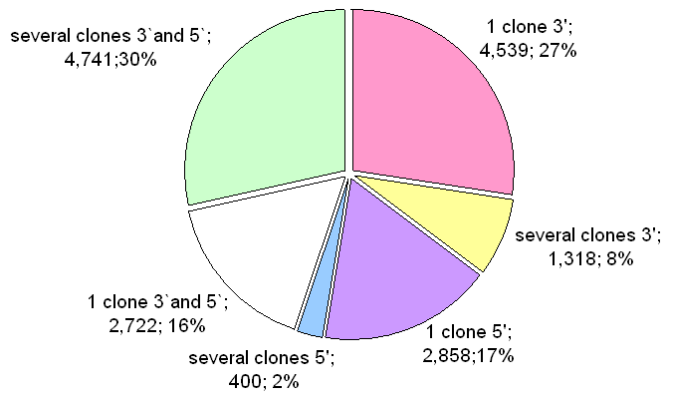
Composition of white spruce consensus sequences (contigs and singletons) according to orientation of direction of the reads (3' or 5') and according to their redundancy in the database (number of clones).

The assembly of the 49,101 quality reads resulted in 9,354 contigs and 7,224 singletons, representing a total of 16,578 consensus sequences named ArboreaSet in the following. As a result of our sequencing strategy, 46% of the consensus sequences were derived from overlapping 3' and 5' reads of one or more cDNA clones (Figure 1). We considered that non-overlapping 3'and 5' reads derived from the same cDNA clone (i.e. a spanning clone) belonged to the same transcript. We thus used spanning clones to link several consensus sequences and obtained a reduced set of 14,471 sequences that we defined as "transcripts".

**Figure 2:**
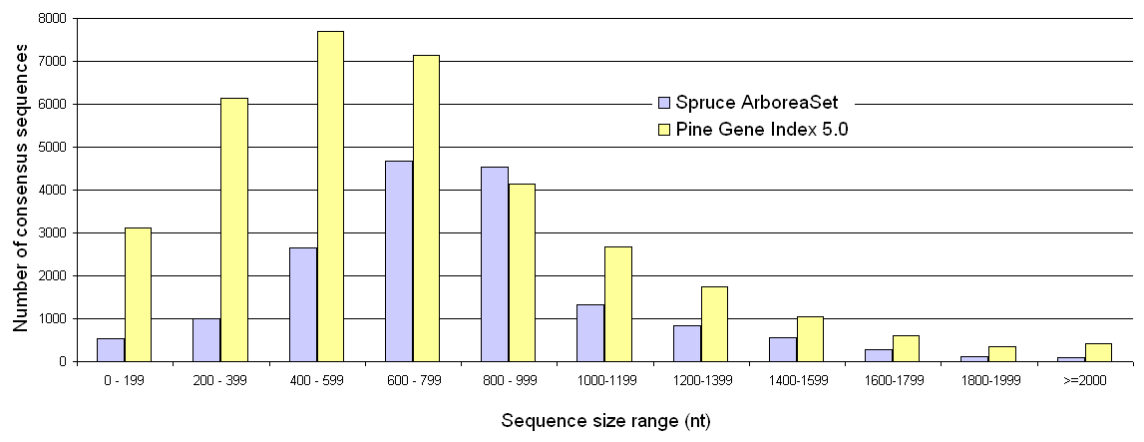
**Sequence sizes**. Size distribution of the consensus sequences derived from the pine (PGI5.0) and white spruce (ArboreaSet) assemblies.

The proportion of consensus sequences represented by more than one cDNA clone was only 39%, which provides an estimate of the sequencing redundancy. The bidirectional sequencing strategy and the average length of quality reads (Table 1) also impacted upon the length distribution of consensus sequences. The most striking feature of the set of spruce consensus sequences is the small proportion of sequences under 600 nucleotides compared to the PGI5.0 pine sequence assembly (mainly derived from 5' reads) despite its much larger number of sequences (Figure 2). The average consensus lengths were 797 and 690 nucleotides, for the Arborea and PGI5.0 sets, respectively; the median lengths were 784 and 612 nucleotides for these same datasets. The deepest contigs in the ArboreaSet included sequences homologous to genes coding for a DNA methylase (202 clones), the translation elongation factor-1 alpha (111 clones), a polyubiquitin (68 clones), an homocysteine methyltransferase (68 clones), a S-adenosylmethionine synthetase (65 clones).

We also estimated the level of redundancy among the 16,578 consensus sequences by comparing the entire set of sequences to itself with the *blastn *program (Table 2). High scoring pairs revealing more than 98% of identity over more than 100 bp were used to define 13,686 contig groups, indicating a level of 21.1% of redundancy among the consensus sequences (Table 2). The very large majority of the 13,686 contig groups were comprised of one or two consensus sequences; however a few groups (3) were made up of more than ten distinct sequences. In a collection of 43,141 consensus sequences derived from 260,000 sugarcane ESTs, the redundancy was estimated at 22%, based upon 98% over 100 bp [29]. In a *Citrus *EST sequence assembly, the level of redundancy was estimated at 25% [30]. Overall the redundancy is in the same range as observed in other projects conducted in mouse [31] or honey bee [32].

**Table 2:** Contig groups according to several levels of sequence identity based on 100 nt of overlap

Number of contigs per group	90%	96%	98%	99%
1	10,036	10,997	11,767	13,295
2	1,576	1,422	1,377	1,083
3	443	386	341	210
4	175	153	103	61
5	93	72	48	22
6	52	40	26	6
7	15	17	10	2
8	13	8	7	1
9	10	1	3	1
≥10	21	12	3	3
Total number of groups	12,435	13,109	13,686	14,685

Spruce and pine EST datasets are populated with allelic variants for many loci because conifers are outbred and highly heterozygous. As a consequence, the number of genes sampled may be estimated more or less accurately from the number of contigs or contig groups, depending upon the parameters that are used for their assembly and clustering. To our knowledge, the impact of assembly parameters has not been directly assessed in conifers or other Gymnosperms. On the other hand, the average nucleotide diversity was reported to be low for conifers [24,26]; for example, sequence variation was estimated in pines with the average mutation population parameter ? = 0.00407 in *Pinus taeda *[33], ? = 0.00241 in *P. pinaster*, ? = 0.00186 in *P. radiata *[34] and ? = 0.0013 in *P. sylvestris *[35]. These data suggest that the use of stringent criteria were appropriate for the assembly (into contigs) of the spruce sequence dataset comprised in part of allelic sequences. We also defined contig groups with less stringent criteria aiming to evaluate sequence redundancy. We recognize, however, that some contigs may contain paralogs, especially for slow-evolving gene families as discussed in other reports on plant EST clustering [29,30]. For these reasons, the contig groups are thought to provide a conservative estimate of the number of genes, i.e. the minimum number of genes sequenced.

### Sequence comparisons with other species

**Figure 3:**
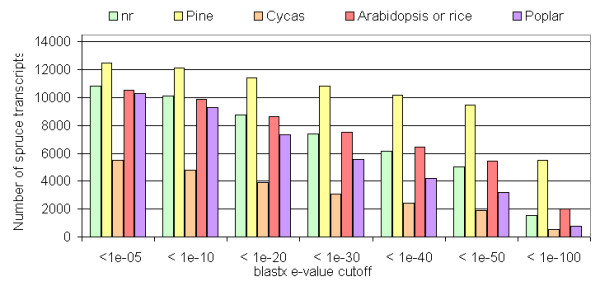
**Sequence similarities**. Number of white spruce transcript sequences similar to Uniref100 proteins, *Arabidopsis*, pine, *Cycas *according to the blast e-value cutoff.

We performed sequence similarity searches using *tblastx *and *blastx *to compare the ArboreaSet to several sequence datasets from Angiosperms (*Arabidopsis*, rice, poplar) and Gymnosperms (*Cycas *and pine), and to the Uniref100 protein database for several e-value cutoffs (Figure 3). In the following sections, the data were obtained with an e-value cutoff of 1e-10 unless specified otherwise.

#### Sequence comparisons with the pine database and Angiosperm genomes

We found that 84.4% the Arborea transcript set (12,108 transcripts) showed sequence similarity with a contig of the Pine Gene Index (PGI5.0) which contains the largest assembly of publicly available pine ESTs (Figure 3). All of the *tblastx *searches detected a greater number of matches with PGI5.0 than with the Uniref100 protein database, in which the PGI5.0 consensus sequences are not represented. We examined whether the lack of similarity of the remaining 15.6% spruce transcripts (with no counterpart in the pine database) could be attributed to the non overlap of pine and spruce contigs derived from 5' and 3' sequences, respectively. More than half of the non matching spruce transcripts (9.8% of the total transcripts) were indeed derived only from 3' reads. Therefore, the lack of similarity of many of the sequences is not sufficient to conclude whether a pine homolog is absent from the database. Nonetheless, 6.6% of the spruce transcripts were derived from 5' reads alone (predominantly) or both 5' and 3' reads and, did not match a pine contig. For these sequences, there is a high likelihood that a similar pine transcript has not been sequenced thus far.

As might be expected, the overall sequence similarity was lower with Angiosperms than pine sequences. There were fewer matches and the number of matches decayed more rapidly as we used more stringent e-value cutoffs with *tblastx *against Angiosperms sequences. At the protein level, 68.4% (9,898) of spruce transcripts matched a sequence from *Arabidopsis *or rice with a *tblastx *e-value < 1e-10 and the proportion dropped to 37.6% for highly conserved sequences (e-value < 1e-50) compared to 65.3% with pine. A similar trend was observed with the poplar genome sequence which gave slightly lower similarities than *Arabidopsis *and rice sets, i.e. 64.3% and 21.6% matches with e-values below 1e-10 and 1e-50, respectively (Figure 3).

#### Complementarity of the sequencing projects in several species

**Figure 4:**
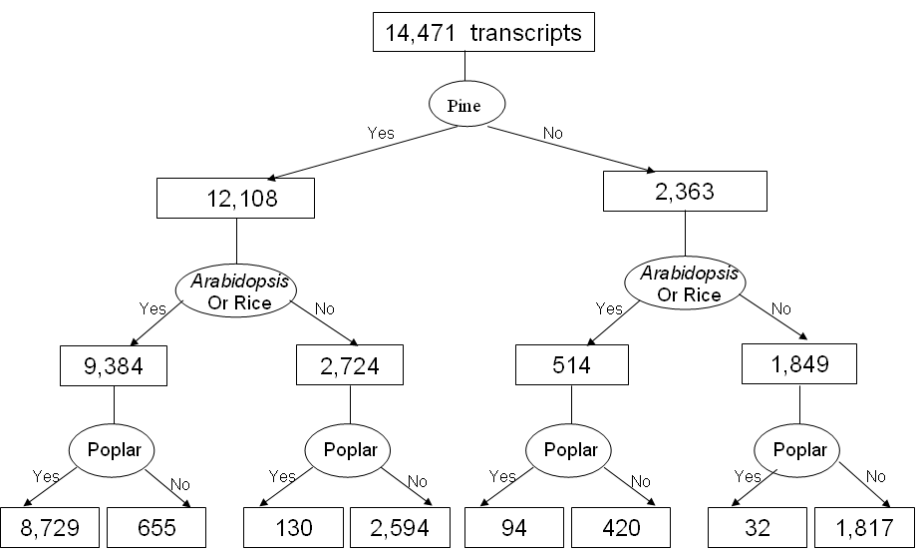
**Hierarchical presentation of the number of spruce transcripts with or without similarities with pine, *Arabidopsis*, rice and poplar**. The numbers were derived by the filtering of *tblastx *searches with an e-value < 1e-10.

We analyzed and compared the overlap of sequence datasets derived from spruce, pine, *Arabidopsis*, rice and poplar, to develop an overall understanding of the complementarity between the sequencing projects in these diverse species. The extent of the overlap based upon *tblastx *matches is shown in Figure 4. In total, 77.5% transcripts found both in pine and spruce databases (9,384 of 12,108) gave a match with *Arabidopsis *or rice. However, only 514 (3.6%) spruce transcripts without any homolog in the pine database had a homolog in *Arabidopsis *or rice. In contrast, 41.7% out of the 26,616 consensus sequences from PGI5.0 that had no match in the ArboreaSet, gave a hit in *Arabidopsis *or rice. These sequence results appear consistent with the extent of divergence that might be expected between the genomes of Gymnosperms and Angiosperms. In a previous study, pine consensus sequences gave 61.5%, 59.4% and 55% matches against *Arabidopsis*, rice and poplar, respectively [9]. With the same similarity search parameters, the spruce transcripts – which contains longer sequences on average – gave slightly more matches against *Arabidopsis *or rice (68.4%), and as well as against poplar (64.3%).

Comparisons to the poplar genome sequence gave fewer matches and only a small number of matches not identified with *Arabidopsis *or rice (Figure 4). Only 89.1% of the spruce transcripts (8,823 out of 9,898) that matched an *Arabidopsis *or rice sequence also had a similarity to a sequence in the poplar genome. Furthermore, 3.5% of the spruce transcripts which lacked similarity to *Arabidopsis *or rice gave a match against the poplar genome. In the end, sequence similarity searches against the poplar genome only allowed us to annotate 162 additional sequences (0.7% of the spruce transcripts) compared to data derived from comparisons with *Arabidopsis *or rice. Such a trend is expected given the relatively close proximity between poplar (Salicaceae) and *Arabidopsis *(Brassicaceae).

The results indicate that data derived from Angiosperms species alone are insufficient for annotating sequences in conifers and that computational tools specifically developed for Gymnosperms are needed to help recognize functional regions in sequences like coding sequences or motifs in around 30% of conifer sequences with no obvious counterpart in Angiosperms. For example, the software *Diogenes *for predicting open reading frames in sequences was trained based on Pinaceae derived sequences for this purpose [36].

### Functional annotation

In total, 10,130 (70%) of the spruce transcripts displayed matches to proteins of known or unknown function, based on the *blastx *analysis against the Uniref100 database. We conducted Hidden Markov Model (HMM) searches against the PFAM protein family database [37,38] to evaluate the proportion of the spruce transcripts homologous to families with an assigned function. Overall, we found that 52% of the 14,471 spruce "transcripts" showed similarity with 1,655 PFAM protein families (p-score below 1e-10). There were 157 of these PFAM families annotated as "DUF, Domain of Unknown Function", which showed similarities with 488 transcripts, and 20 families annotated as "UPF, Uncharacterized Protein Family" showing similarities with 45 transcripts. In the end, a total 48% of the spruce transcripts were similar to 1,478 PFAM families when DUFs and UPFs were excluded.

A separate approach using the Gene Ontology scheme [27] categorized 39% of the ArboreaSet contigs into 16 molecular functions based on similarity with functionally annotated genes in other organisms (Table 3). Functional categories were assigned by using the GO terms correlated to similar proteins from Uniref100 [39] or from the *Arabidopsis *databases [40]. In the molecular function category, 39% of the contigs were correlated to a GO term. When the less reliable electronically inferred functional annotations were excluded, 30% of the ArboreaSet contigs were assigned molecular function annotations. The catalytic activity category included the largest number of sequences, followed by the proteins of unknown function. The classification we obtained was similar to that in the PGI5.0 database [41]. A significantly larger proportion of contigs were annotated in spruce than in pine, since we considered all of the *blastx *hits that met the alignment criteria, while the PGI5.0 annotations used only the top hit. Due to the restricted number of well-characterized conifer genes, correlating conifer sequences to Gene Ontology terms relies primarily on conservation with Angiosperms sequences (mainly *Arabidopsis *and rice). Therefore, the GO annotated contigs in spruce and in pine are the ones conserved with Angiosperms.

**Table 3:** Consensus sequences correlated to terms belonging to the "molecular function" categories of the Gene Ontology

Molecular functions	Annotations including electronic annotations			Annotations excluding electronic annotations		
	Number of consensus sequences	% of the number of annotated consensus sequences	% of the total number of consensus sequences	Number of consensus sequences	% of the number of annotated consensus sequences	% of the total number of consensus sequences

Triplet codon-amino acid adaptor activity	0	0	0	0	0	0
Chaperone regulator activity	0	0	0	0	0	0
Motor activity	23	0.35	0.14	3	0.06	0,02
Enzyme regulator activity	47	0.71	0.28	27	0.53	0.16
Nutrient reservoir activity	50	0.76	0.30	4	0.08	0.02
Translation regulator activity	70	1.06	0.42	59	1.16	0.36
Antioxidant activity	73	1.10	0.44	52	1.02	0.31
Signal transducer activity	77	1.16	0.46	33	0.65	0.2
Obsolete molecular function	113	1.71	0.68	76	1.5	0.46
Transcription regulator activity	118	1.78	0.71	73	1.44	0.44
Chaperone activity	166	2.51	1	142	2.79	0.86
Structural molecule activity	283	4.28	1.70	240	4.72	1.45
Transporter activity	503	7.60	3.03	335	6.59	2.02
Binding	1,248	18.87	7.52	741	14.6	4.46
Molecular function unknown	1,340	20.26	8.07	1,340	26.4	8.07
Catalytic activity	2,504	37.85	15.08	1,956	38.5	11.8
Total	6,615	100	39.84	5,081	100	30.6

**Figure 5:**
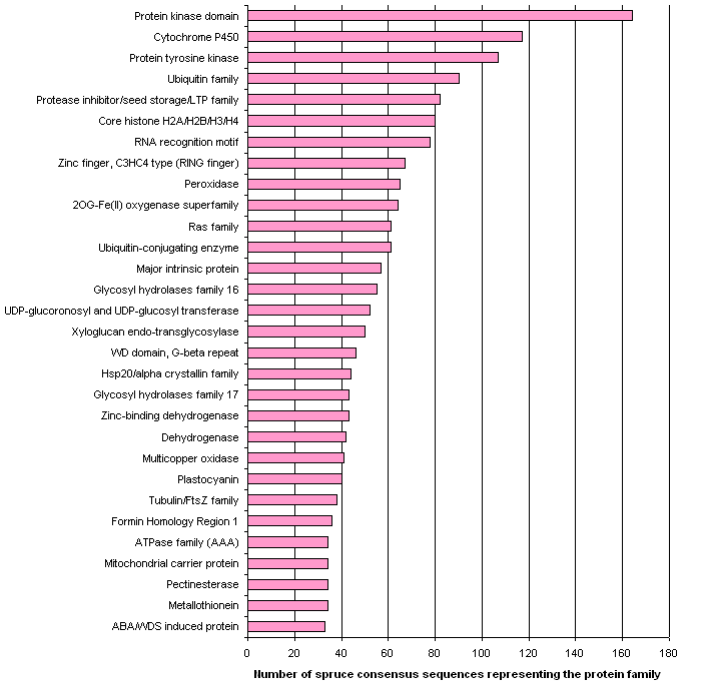
**Protein families**. Occurrence of the 30 most abundant protein families in the white spruce dataset identified by HMM searches with an e-value < 1e-10 against the PFAM database.

HMM searches against the PFAM database showed that the most abundant sequences in plant genomes were also among the most represented in the ArboreaSet (Figure 5). Highly comparable findings were made with the pine dataset (PGI5.0). A similar analysis conducted with the sugarcane SUCEST database indicated that the most abundantly represented molecular functions were largely overlapping between conifers and sugarcane [29].

#### Families of putative transcription factors

We identified putative transcription factors based upon the assignment of GO terms, as well as sequence comparison to PFAM domains and families [37,38]. The GO based annotation "transcription regulator activity" was assigned to 113 spruce sequences (including 40 assignments based upon automatic annotations) and the annotation "transcription factor activity" (GO:0003700) was assigned to 90 of the same consensus sequences. We also conducted HMM searches with the 41 PFAM profiles representing the plant transcription factors described in the *Arabidopsis thaliana *Transcription Factor Database (AtTFDB, from the *Arabidopsis *Gene Regulatory Information Server, AGRIS) [42] and identified 304 spruce transcripts (Table 4). Only 43 of these putative transcription factors were identified by both approaches. The combined total represented 388 putative transcription factors sequences. The most frequent sequence similarities were with C3HC4 zinc finger domain, WD, and AP2, respectively.

**Table 4:** Identification of transcripts encoding putative regulatory proteins. Sequences were identified based on HMM searches suported by p-score < 1e-10 with PFAM profiles available for families of regulatory proteins. The PFAM accessions for which no homology was found in SpruceDB through HMM search were not reported.

Protein family	PFAM accession	Number of spruce transcripts
Zinc finger, C3HC4 type (RING finger)	PF00097	66
WD, G-beta repeat	PF00400	44
AP2 domain-B3 DNA binding domain	PF00847	19
HMG (high mobility group) box	PF00505	16
MADS Family – SRF-type transcription factor – K-box region	PF00319	14
MYB DNA-binding	PF00249	13
AUX/IAA	PF02309	12
Histone-like transcription factor (CBF/NF-Y) and archaeal histone	PF00808	11
PHD finger – CW-type Zinc Finger	PF00628	10
No apical meristem (NAM) protein	PF02365	10
GRAS Family	PF03514	10
WRKY DNA-binding domain	PF03106	9
NAC domain	PF01849	9
Homeobox domain	PF00046	8
bZIP transcription factor – bZIP Maf transcription factor-G-box binding protein MFMR	PF00170	8
B-box zinc finger	PF00643	6
TUB Family	PF01167	6
Helix-loop-helix DNA-binding domain – Myc amino-terminal region	PF00010	5
KNOX2 domain	PF03791	3
LIM domain family – PET Domain	PF00412	5
Dof domain, zinc finger	PF02701	4
GATA zinc finger	PF00320	3
TCP family transcription factor	PF03634	2
CCAAT-HAP2 Family CCAAT-binding transcription factor (CBF-B/NF-YA) subunit B	PF02045	2
SBP (Sqamosa-promoter binding protein) floral development	PF03110	1
HSF Family (Heat shock protein promoter binding)	PF00447	1
EIL Family ethylene insensitive 3	PF04873	1
B3 DNA binding domain	PF02362	1
ARID/BRIGHT DNA binding domain – ELM2 domain	PF01388	1

#### Cell wall related genes

Many of the libraries that we constructed were derived from secondary vascular tissues from stems or roots, or from whole stems or roots containing primary as well as secondary vascular regions. Therefore, we aimed to classify genes which encode proteins potentially involved in cell wall assembly. As a first step toward this goal, our collection of spruce transcripts was blasted against the sequences from the Cell Wall Navigator Database [43] [see Additional file 2], comprised of proteins involved in primary cell wall structure and assembly. In total, we found that 708 spruce contigs were similar to sequences of cell wall related proteins, with nearly all of the subclasses represented. We also searched for genes encoding enzymes involved in the biosynthesis monolignol precursors based upon sequence similarity with the set identified in *Arabidopsis *by Reas *et al*. [44], and identified 47 additional contigs (Supplemental data 2).

#### Redundancy analysis suggests larger size of selected protein families in spruce compared to Angiosperms

**Figure 6:**
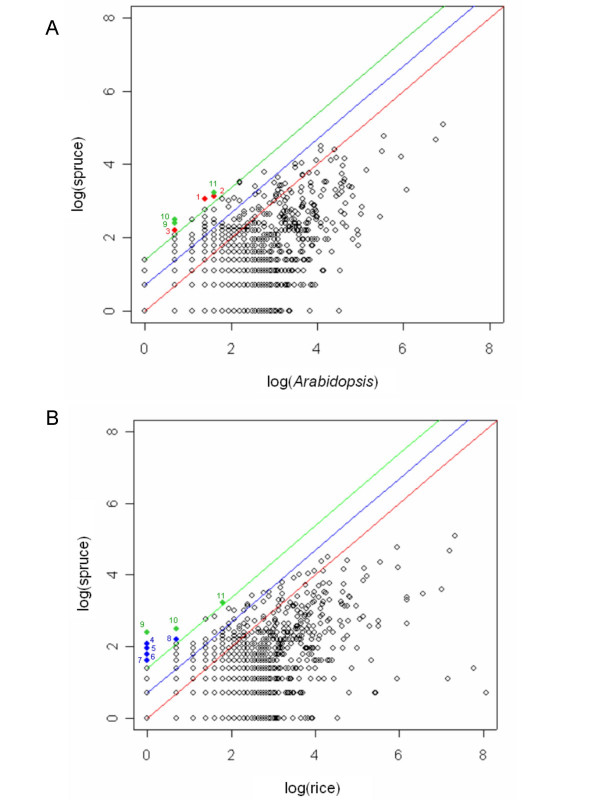
**Number of spruce consensus sequences (identified by HMM searches against PFAM) relative to the size of the gene families in *Arabidopsis *(a) and rice (b)**. Each point represents a protein family detected by the HMM searches with p-score < 1e-10. Point coordinates are the number of genes found in the analysed Angiosperm genome (x axis) and the number of contigs found in the spruce database (y axis), after a log transformation. The red, blue and green lines represent the ratios 1:1, 1:2, and 1:4, respectively. Red points represent sequences found 4 times more in white spruce than in *Arabidopsis*: 1. AWPM-19-like family [PF05512], 2. Chalcone and stilbene synthases, C-terminal domain [PF02797], 3. Phosphoenolpyruvate carboxykinase [PF01293]. Blue points represent sequences found 4 times more in spruce than in rice : 4. Ribosomal protein S28e [PF01200], 5. Cyclin-dependent kinase regulatory subunit [PF01111], 6. TIR domain [PF01582], 7. Splicing factor 3B subunit 10 [PF07189], 8. Ribosomal Proteins L2, C-terminal domain [PF03947]. Green points represent sequences found 4 times more in spruce compared to both *Arabidopsis *and rice: 9. Translationally controlled tumour protein [PF00838], 10. S-adenosyl-L-homocysteine hydrolase [PF05221], 11. S-adenosylmethionine synthetase, C-terminal domain [PF02773].

It is not expected that Gymnosperm genomes will be sequenced in the foreseeable future, therefore we undertook a preliminary comparative analysis of protein families using the 14,471 spruce transcripts, to assess whether insights may be gained into the relative size of protein families in Gymnosperms and Angiosperms. We compared the occurrence of proteins in the ArboreaSet to that observed in the *Arabidopsis *genome (1,611 families) as well as in the rice genome (1,601 families) identified with HMM searches against the PFAM database. As might be expected from the partial coverage of the spruce genome, the vast majority of the protein families were represented by a larger number of sequences in the *Arabidopsis *and rice genomes than in ArboreaSet (Figure 6). However, several families gave twice as many hits in the ArboreaSet (67 and 58 families compared to *Arabidopsis *and rice, respectively) and a few families had at least 4 times more sequences (6 for *Arabidopsis *and 10 for rice, including 3 families for both). Some of these families encoded proteins that can be linked to the cell wall catabolism (PF01476), single carbon metabolism (S-adenosylmethionine synthetase PF02773), the cytoskeleton (Translationally Controlled Tumour Protein, TCTP family PF00838) or the cellular membrane (AWPM-19-like family PF05512). We verified that the size of the 4-fold larger families of spruce sequences was not inflated due to incomplete assembly of 3' and 5' reads. For two of the putatively larger families, we examined the protein and nucleic acid sequence diversity between the different consensus sequences in order to estimate the number of family members (results presented below).

#### The cytoskeleton related TCTP family

The translationally controlled tumour proteins (TCTPs) are anti-apoptotic proteins, named for their preferential synthesis in the early phase of some tumours [45]. They are implicated in both cell growth and division and have been shown to bind to tubulin in the cytoskeleton. In plants, similar proteins were identified in alfafa [46] and *Pharbitis mil *[47].

The TCTP domain (accession : PF00838) was found in only two *Arabidopsis *sequences (At3g16640.1 and At3g05540.1) and one rice sequence (location in Gramene: LOC_Os11g43900.1). In contrast, there were 11 transcripts in the ArboreaSet that encompassed a highly conserved region of TCTPs and showed a high level of sequence conservation with *Arabidopsis *TCTPs (e.g. 70% a.a. identity for predicted sequence of Contig9531 and the *Arabidopsis *sequence gb|AAM66134.1). In total, 8 of the 11 spruce TCTP transcripts encompassed a putative complete coding sequence that overlapped with the *Arabidopsis *proteins. Pairwise nucleic acid sequence comparisons of the 11 spruce transcripts were used to identify 5 distinct sequence groups, likely representing a minimum of 5 different genes (Table 5). Based upon these data, the TCTP family provided an example of putative differential protein family size between spruce and the Angiosperms represented by rice and *Arabidopsis*.

**Table 5:** Pairwise comparison of white spruce consensus sequences related to the translationally controlled tumour proteins (TCTP). Nucleic acid identities were determined using the Smith-Waterman algorithm (*water*) available in the *EMBOSS *suite [71] in a 138 bp region of the 5' UTR immediately upstream of the first codon (ATG), (above the diagonal); and, along the complete sequence of the consensus sequences (under the diagonal). The diagonal shows the contig length.

	Sequence10076	Sequence10707	Sequence9531	Sequence7749	Sequence1882
Sequence10076	805	88/162 (54.3%)	54/84 (64.3%)	70/159 (44.0%)	83/144 (57.6%)
Sequence10707	761/890 (85.5%)	977	111/157 (70.7%)	71/147 (48.3%)	99/154 (64.3%)
Sequence9531	759/889 (85.4%)	925/1034 (89.5%)	1124	65/133 (48.9%)	101/159 (63.5%)
Sequence7749	515/659 (78.1%)	548/736 (74.5%)	596/938 (63.5%)	945	73/147 (49.7%)
Sequence1882	719/815 (88.2%)	742/823 (90.2%)	750/906 (82.8%)	523/687 (76.1%)	796

#### The SAMS family

Sequences encoding S-adenosylmethionine synthetases (SAMS), a family of enzymes involved in single carbon metabolism and in lignin precursor biosynthesis [48] were represented by 24 consensus sequences encompassing at least seven spruce genes (Table 6). It has been reported that *sams *genes belong to small gene families in other plant species [49-53]. In *Arabidopsis*, four *sams *genes were identified. In rice, three sequences encoding complete proteins of 396 amino acids were found, as well as two sequences encoding truncated proteins of 164 amino acids [54].

**Table 6:** Pairwise comparison of white spruce consensus sequences related to the S-adenosylmethionine synthetase (SAMS). Nucleic acid identities were determined using the Smith-Waterman algorithm (*water*) available in the *EMBOSS *suite [71] in a 99 bp region of the 3' UTR immediately downstream the stop codon (above the diagonal) and along the complete sequence of the consensus sequences (under the diagonal). The diagonal shows the contig length.

	Sequence 10446	Sequence 10482	Sequence 10630	Sequence 10683	Sequence 10828	Sequence 8600	Sequence 9676
Sequence10446	1677	46/97 (47.4%)	48/113 (42.5%)	51/117 (43.6%)	45/85 (52.9%)	85/106 (80.2%)	44/98 (44.9%)
Sequence10482	1096/1607 (68.2%)	1467	54/78 (69.2%)	65/92 (70.7%)	50/84 (59.5%)	49/114 (43%)	45/96 (46.9%)
Sequence10630	1126/1641 (68.6%)	1343/1557 (86.3%)	1540	69/113 (61.1%)	48/78 (61.5%)	47/95 (49.5%)	55/111 (49.5%)
Sequence10683	1143/1711 (66.8%)	1342/1521 (88.2%)	1357/1582 (85.8%)	1531	49/103 (47.6%)	58/116 (50%)	46/117 (39.3%)
Sequence10828	1202/1814 (66.3%)	1262/1534 (82.3%)	1343/1714 (78.4%)	1306/1604 (81.4%)	1679	49/95 (51.6%)	49/109 (45%)
Sequence8600	1349/1691 (79.8%)	1058/1536 (68.9%)	1089/1532 (71.1%)	1092/1583 (69%)	1120/1656 (67.6%)	1476	41/71 (57.7%)
Sequence9676	1025/1418 (72.3%)	1314/1459 (90.1%)	1276/1397 (91.3%)	1261/1381 (91.3%)	1179/1369 (86.1%)	1026/1462 (70.2%)	1356

We analyzed the 8 spruce *sams *transcripts that encompassed complete protein coding sequences averaging 393 amino acids in length. The predicted proteins were very highly conserved with Angiosperm SAMS. For example, the *Arabidopsis *SAMS2 protein (locus At4g01850) had a similarity of 88% (345/390a.a) and 90% (354/390 a.a) with the predicted proteins from the spruce contigs 10446 and 10482, respectively. Pairwise comparisons of the spruce coding sequences showed they are highly conserved, yet they could be divided into seven groups of sequences with 66.3% to 91.3% identity (Table 6). We also analyzed the nucleic acid sequence of their 96 bp 3' UTR and found significant variability between groups, with sequence identities varying from 42.5% to 70.7% (Table 6). These results provided a strong indication that these putative *sams *transcripts represented 7 distinct genes. Protein and nucleic acid sequence comparisons supported the hypothesis that the SAMS proteins form a larger family in the spruce genome than in *Arabidopsis *and rice. In rice, the presence of two pseudogenes indicated that protein family expansions through duplication events have been followed by gene loss during the evolution. Two *sams *genes were described in *Pinus contorta *[55]; however, large-scale EST sequencing in *Pinus taeda *[56] identified 16 consensus sequences, suggesting that the relatively large family size of SAMS in spruce may also apply to pine and other Gymnosperm genomes.

### Development of Spruce DB

The relational database, SpruceDB, was created to allow complex queries into the spruce ESTs, assembled consensus sequences and results of similarity analyses. The database can be accessed via web browser [28]. Web-based tools provide facilities for exploration of this information resource. The ESTs or contigs can be retrieved based on library composition and sequence similarities. Web links from the database query pages retrieve the actual EST and contig sequences from the Biodata web pages [56].

#### Structure and data sources

**Figure 7:**
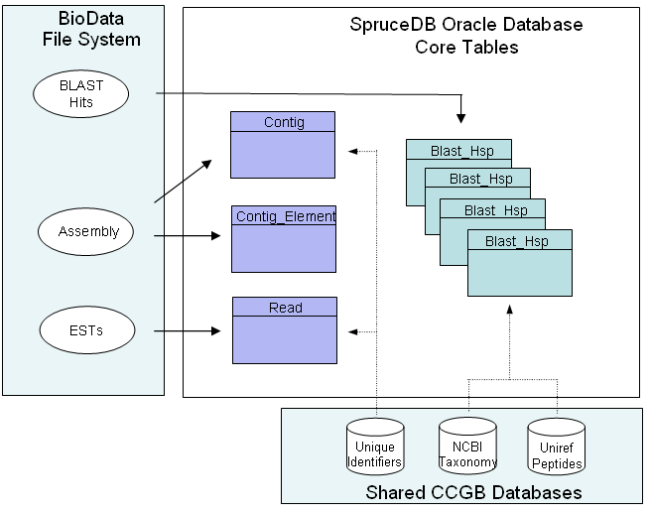
**SpruceDB core tables and data sources**. Data from flat files on ESTs, Assemblies and *blast *hits is loaded into the core tables Read, Contig, Contig_Element and Blast_Hsp. Additional information on taxonomy identifiers and Uniref100 peptides is obtained from shared databases.

The database schema for SpruceDB is identical to the one successfully used by the MtDB2.0 database for *Medicago truncatula *EST data [57]. SpruceDB is hosted on a Sun V880 server running the Oracle 8i Database Management System. The data sources and core tables for the database are illustrated in Figure 7. Sequence trimming methods and assembly parameters for *Phrap *are described in the Methods section. Information about ESTs and consensus sequences assembled with *Phrap *is extracted from flat files and loaded into the core tables Read, Contig and Contig_Element. These tables store the sequences and lengths, base qualities, EST libraries, clone names, and assembly name. Several tables store pre-computed *blast *hit information from blast similarity analyses against several target databases: UniRef100 peptides, *Arabidopsis *proteins, SpruceDB itself. Data fields include analysis program, target name, hit identifier, e-value, identities and taxonomy identifier for each hit. All *blast *hits with e-values less than 0.01 are loaded into the database.

#### Interface

**Figure 8:**
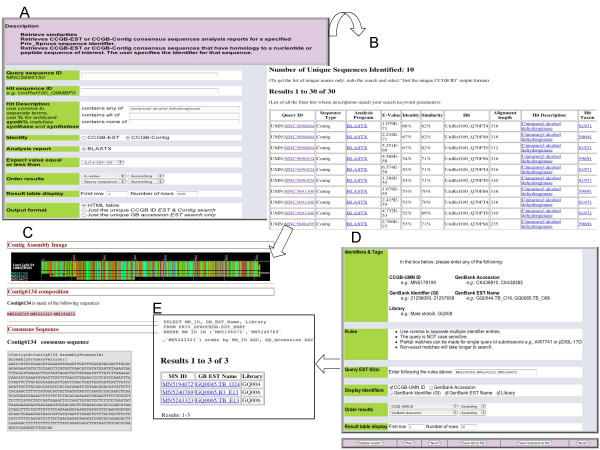
**Examples of the interface of the SpruceDB database**. A) Use of Query 1 to search for contigs matching "cinnamoyl alcohol dehydrogenase" among the blastx results loaded in the database. B) Display of the results indicating alignment parameters (alignment length, similarity and identity level). C) BioDATA page linked to by clicking on MNC5693153 in Query 1 results. The upper figure illustrates the alignment of the members of the contigs in a color coded manner. Read names written in blue and white color refer to 5'and 3'reads, respectively. D) Query 8 allowing to retrieve sequence aliases and library names for specified MN_Ids. E) Query 8 results showing libraries GQ004 and GQ006.

The web pages used to query the database allow retrieval of ESTs or contigs based on the cDNA libraries and *blast *hits (Figure 8). Since the nine query pages consist of check boxes and pull-down menus, no programming or knowledge of SQL is required, yet users can generate complex queries. Query 1 retrieves consensus sequences that have *blast *hits containing user-specified keywords or accession numbers. Queries 2–7 are library filter queries which retrieve ESTs or consensus sequences containing "any of", "all of", or "only" ESTs from user-specified cDNA libraries. Queries 3–7 contain taxonomy and e-value filters which retrieve sequences that have *blast *hits to organisms from specified taxa such as "all pines", "all poplars", or *Arabidopsis*. Query 5 combines the library, taxonomy and e-value filters in a single web page. Query 8 retrieves EST sequences using different names (aliases). Query 9 compares consensus sequences between different assemblies.

## Conclusion

In this report, we described a new conifer EST resource derived from 49,101 high quality 5' and 3' reads that were assembled to produce 16,578 consensus sequences averaging 797 nucleotides in length, and representing 14,471 different "transcripts". We estimated the sequencing redundancy at 39% based on the number of consensus sequences represented by more than one cDNA clone. Comparison of the spruce sequences to public sequence datasets from Angiosperms and pine showed that approximately 70% of the sequences had similarity with *Arabidopsis*, rice or poplar sequences, but 84% matched a pine sequence. The majority of the sequences that did not give a match in pine did not produce a match with any of the Angiosperms either. We used a variety of approaches based on sequence similarity searches to assigned putative functions to the ArboreaSet sequences, including *blast *searches against general and specialized datasets, GO term assignation, HMM searches against PFAM protein families and domains. These analyses were used for the systematic identification of diverse putative transcription factors, cell wall related enzymes and structural proteins, and revealed a few protein families that are thought to be larger in spruce than in the well-characterized genomes of *Arabidopsis *and rice.

These comprehensive analyses to enable the annotation of spruce sequences provide critical information to help identify target genes for functional analysis and association studies. Studies are now being planned based on these data to search for DNA polymorphisms underlying the extensive phenotypic variation which occurs in natural and breeding populations. These studies will focus on sequences encoding proteins relevant for adaptation, growth and wood formation for large-scale SNP discovery and genotyping required for association studies and gene mapping. It is therefore essential that we develop databases of annotated coding sequences so that we may rapidly identify and screen the most suitable targets. As a first step toward this goal, the relational database SpruceDB was created to allow complex queries into the spruce ESTs, assembled consensus sequences and results of similarity analyses. By using this EST resource, we have also developed a low redundancy cDNA microarray comprised of 9,690 sequences, which, in combination with multiple sequence annotations, will be a powerful tool to investigate transcriptome modulation in spruces and conifers.

## Methods

### Plant material

All of the libraries were comprised of a single organ or tissue, and the majority of libraries were developed by pooling samples collected at different points along a time course, along the diurnal cycle, at several stages of differentiation or from different treatments (Supplemental data 2 and [58]). Treatments known to affect plant physiology were applied to saplings (young trees) aiming to stimulate different transcript profiles. These treatments included N and P fertilization as well as stem girdling. Three libraries were made from whole root systems of very young spruce seedlings, produced through tissue culture, grown in sterile growth media. Most of the libraries were derived from one genotype (pg-653), however four libraries were comprised of two or more genotypes. The secondary xylem collected from saplings (library GQ007) was comprised of the entire sampling of woody tissues collected from seedlings; however, only the differentiating partly-lignified secondary xylem was collected from mature trees as previously described [16]. The secondary xylem tissues were collected by first gently separating the bark from the underlying wood and scraping the soft tissues inward of the cambial area. The secondary phloem of mature trees was collected by gently scrapping the inner surface of the bark with a scalpel blade. All tissue samples were frozen in liquid nitrogen and then stored at -80°C until RNA extraction immediately upon removal from the tree, seedling or tissue culture vessel.

### cDNA library construction, quality controls and high-throughput sequencing

We began the construction of each library with 1000 micrograms of total RNA or more, isolated using the method of Chang *et al*. [59]. Poly A+ RNA was isolated using the PolyATtract mRNA Isolation System (Promega, San Luis Obispo, CA, USA). The polyA+ RNA was treated with methylmercury hydroxide according to the manufacturer's instruction (Stratagene, La Jolla, CA, USA) to relax its secondary structure. Double-stranded cDNA was synthesized from 5 micrograms (µg) of poly A+ selected RNA using a pBluescript II SK (+) XR cDNA Library Construction kit (Stratagene, La Jolla, CA, USA). The reverse transcription step was carried out with either Superscript II or Superscript III and StrataScript (Invitrogen, Burlington, ON, Canada; Stratagene, La Jolla, CA, USA) as described in the manufacturer's instructions. The double stranded cDNAs were fractionated using the Drip column method (Stratagene, La Jolla, CA, USA) or by agarose gel electrophoresis on NuSieve GTG Agarose (Mendel, Guelph, ON, Canada) followed by selective elution of particular-sized cDNA molecules by ß-Agarase I digests according to the manufacturer's instruction (NEB, Pickering, ON, Canada). The size distribution of the resulting double cDNA synthesized in second-strand fractions was visualized by electrophoresis on a 1.4% alkaline agarose gel [60]. The fractions of 600 pb to 1.2 kb and above 1.2 kb were selected, pooled, directionally ligated into the EcoRI and XhoI restriction sites of the pBluescript II SK (+) XR vector (Stratagene, La Jolla, CA, USA), and transformed into E.coli DH10B competent cells (Invitrogen, Burlington, ON, Canada) by electroporation. The library quality assessment used test ligations to determine library titer. We also estimated the proportion of empty vectors as based upon the proportion of blue to white colonies grown on LB agar supplemented with X-GAL/IPTG (Table 1). The average cDNA insert size was determined by PCR screening of 48 to 96 random white colonies (assumed contain plasmids with inserts) per test ligation, followed by determination of the PCR product size by gel electrophoresis. The highest quality libraries were those estimated to have the highest proportion of inserts above 1.6 Kb (Table 1). High-throughput sequencing of libraries was completed using standard methodsas described by Yang *et al*. [61].

### EST processing and assembly

Sequence traces from the spruce EST libraries were analyzed with the *Phred *base calling software (version 0.980904) to generate raw sequences [62]. Peaks with *Phred *quality values less than 20 were considered to be ambiguous and were assigned base N. Quality trimming and vector filtering (with polyA/polyT removal, as appropriate) were done. Processed sequences were then assembled using the base quality files and *Phrap *(version 0.990329) [63]. *Phrap *contigs were evaluated for chimeric sequences, and reassembled after removing chimeric reads. The *Phrap *assembly parameters used were minmatch 50 and minscore 100. Only reads with at least 100 nt of sequence with a quality score above 20 were assembled. EST sequences were submitted to dbEST at the National Center for Biotechnology Information [64] under accession numbers : [Genbank:CK434215-CK445169] and [Genbank:CO472624-CO490610].

### Quality control of consensus sequences

The quality control of resulting consensus sequences used a system developed at the CCGB. This system uses information that is included in the contig *ace *file generated by *Phrap*. From the *ace *file, several important characteristics of a consensus sequence and its member sequences can be determined. The first characteristic used in this process is the "shape" of the consensus sequence, or how the assembled reads overlap each other. This can be thought of as the profile of the consensus sequence member distribution. Consensus sequences are classified as being of block, staircase, or dumbell shape. Contigs with a dumbell shape are candidates for additional evaluation.

Reads within a dumbell shaped contig are evaluated for their similarity to the consensus sequence of the contig. *Phrap *provides information on the quality regions of assembled sequences, which is used for this step. If the high quality region of the read (as defined by the *Phrap *ace file) has less than 95% consistency with the consensus sequence of the contig, or has more than 5 mismatched bases relative to the consensus, the read is flagged as a suspected chimera, provided it also shows evidence of either a polyA or polyT region.

The final step of the quality control process is to examine the flagged reads visually to find chimeric qualities. Chimeric reads are selected and removed based on their similarity to the consensus sequence and to the individual reads in the contig. A chimeric read may also be indicated if *blast *hits to different proteins are found to be adjacent in the read. The process of chimera detection and removal is often repeated numerous times before arriving at a finished assembly.

### Sequence comparison and assignment into functional categories

Similarity searches were performed with the *tblastx *or *blastx *programs [65] against the TIGR Gene Indices available for *Arabidopsis *(AGI11), rice (OGI16) and pine (PGI5.0), retrieved from the TIGR web site [66] and against one *Cycas *EST assembly [67], retrieved from Sputnik web site [68]. *Blast *searches were conducted against several databases: the NCBI non redundant database (nr), the Uniref100 peptides set [39], and the Cell Wall Navigator Database [43]. HMM searches were conducted with the PFAM profiles (PFAM release16.0) with the local alignment setting since the spruce consensus sequences are fragmentary sequences. The *Arabidopsis *and rice coding sequences were downloaded from the TAIR web site [69] and the Rice Genome Annotation Database from TIGR [70], respectively.

To correlate the spruce consensus sequences to a Gene Ontology (GO) molecular function term, the annotations of homologous Uniref100 and *Arabidopsis *proteins were analysed. For each spruce consensus sequence, the *blastx *hits with a minimum similarity value of 0.75 and a minimum coverage of 0.5 were used in the GO assignment procedure. Similarity was defined as hsp positive/hsp alignment length (hsp : high scoring pair). Coverage was defined as the high scoring pair alignment length × 3/ query length. Among the retained hits, whenever a spruce sequence matched a protein with an associated GO term, this term was transferred to the spruce consensus sequence. Two GO annotation lists were completed: one including evidence codes Inferred from Electronic Annotation (IEA) evidence codes and one excluding IEA evidence.

## Authors' contributions

NP, coordination of bioinformatics activities, data analysis, preparation of the manuscript; CP, LP, JC, JEJ, ER, sequence processing, assembly and annotation, web publishing and database development; MJM, JC, ASé, plant material production, library synthesis, and evaluation; EN, CGC, protein family sequence analyses; YB, SB, GY, JS, ASi, RH, MM, high-throughput EST sequencing and quality assurance; CP, JB, preparation of manuscript; JM, overall project supervision, preparation of manuscript.

## Supplementary Material

Additional File 1 Description of tissues used for cDNA library synthesis: genotype, treatments (type, level and duration), organ, tissue and developmental stage. Click here for file

Additional File 2 **Annotation of proteins related to the cell wall based on similarities with sequences from the Cell Wall Navigator Database **[44]**and lignin biosynthesis enzymes **[45]. Spruce homologs were identified by *tblastx *searches with e-value < 1e-10. Click here for file

## References

[B1] Ahuja MR (2001). Recent advances in molecular genetics of forest trees. Euphytica.

[B2] Dhillon SS, Bonga JM, Durzan DJ (1987). DNA in tree species. Cell and Tissue Culture in Forestry.

[B3] Wakamiya I, Newton RJ, Price JS (1993). Genome size and environmental factors in the genus *Pinus*. Am J Bot.

[B4] Rake AW, Miksche JP, Hall RB, Hanson KM (1980). DNA reassociation kinetics for four conifers. Can J Genet Cytol.

[B5] Ohri D, Khoshoo TN (1986). Genome size in gymnosperms. Plant Syst Evol.

[B6] Murray BG (1998). Nuclear DNA amounts in gymnosperms. Ann Bot.

[B7] Quackenbush J, Cho J, Lee D, Liang F, Holt I, Karamycheva S, Parvizi B, Pertea G, Sultana R, White J (2001). The TIGR Gene Indices: analysis of gene transcript sequences in highly sampled eukaryotic species. Nucleic Acids Res.

[B8] Kirst M, Johnson AF, Baucom C, Ulrich E, Hubbard K, Staggs R, Paule C, Retzel E, Whetten R, Sederoff R (2003). Apparent homology of expressed genes from wood-forming tissues of loblolly pine (*Pinus taeda *L.) with *Arabidopsis thaliana*. Proc Natl Acad Sci USA.

[B9] Pavy N, Laroche J, Bousquet J, Mackay J (2005). Large-scale statistical analysis of secondary xylem ESTs in pine. Plant Mol Biol.

[B10] Guillet-Claude C, Isabel N, Pelgas B, Bousquet J (2004). The evolutionary implications of knox-I gene duplications in conifers: correlated evidence from phylogeny, gene mapping, and analysis of functional divergence. Mol Biol Evol.

[B11] Allona I, Quinn M, Shoop E, Swope K, St Cyr S, Carlis J, Riedl J, Retzel E, Campbell M, Sederoff R, Whetten RW (1998). Analysis of xylem formation in pine by cDNA sequencing. Proc Natl Acad Sci USA.

[B12] Dendrome project. http://dendrome.ucdavis.edu/dfgp/about.html.

[B13] Treenomix project. http://www.treenomix.com.

[B14] Dubos C, Plomion C (2003). Identification of water-deficit responsive genes in maritime pine (*Pinus pinaster *Ait.) roots. Plant Mol Biol.

[B15] Le Provost G, Paiva J, Pot D, Brach J, Plomion C (2003). Seasonal variation in transcript accumulation in wood-forming tissues of maritime pine (*Pinus pinaster *Ait.) with emphasis on a cell wall glycine-rich protein. Planta.

[B16] Egertsdotter U, van Zyl LM, MacKay J, Peter G, Kirst M, Clark C, Whetten R, Sederoff R (2004). Gene expression during formation of earlywood and latewood in loblolly pine: expression profiles of 350 genes. Plant Biol.

[B17] van Zyl L, von Arnold S, Bozhkov P, Chen Y, Egertsdotter U, MacKay J, Sederoff R, Shen J, Zelena L, Clapham D (2002). Heterologous array analysis in Pinaceae: Hybridization of high density arrays of *Pinus taeda *cDNA with cDNA from needles and embryogenic cultures of *P. taeda*, *P. sylvestris *or *Picea abies*. Function Compar Genomics.

[B18] Stasolla C, Belmonte MF, van Zyl L, Craig DL, Liu W, Yeung EC, Sederoff R (2004). The effect of reduced glutathione on morphology and gene expression of white spruce (*Picea glauca*) somatic embryos. J Exp Bot.

[B19] Hertzberg M, Aspeborg H, Schrader J, Andersson A, Erlandsson R, Blomqvist K, Bhalerao R, Uhlen M, Teeri TT, Lundeberg J, Sundberg B, Nilsson P, Sandberg G (2001). A transcriptional roadmap to wood formation. Proc Natl Acad Sci USA.

[B20] Yang J, Park S, Kamdem DP, Keathley DE, Retzel E, Paule C, Kapur V, Han KH (2003). Novel gene expression profiles define the metabolic and physiological processes characteristic of wood and its extractive formation in a hardwood tree species, *Robinia pseudoacacia*. Plant Mol Biol.

[B21] Paux E, Tamasloukht M, Ladouce N, Sivadon P, Grima-Pettenati J (2004). Identification of genes preferentially expressed during wood formation in *Eucalyptus*. Plant Mol Biol.

[B22] Canadian Council of Forest Ministers. http://nfdp.ccfm.org/.

[B23] Florin R (1963). The distribution of conifer and taxad genera in time and space. Acta Horti Bergiani.

[B24] Bouillé M, Bousquet J (2005). Trans-species shared polymorphisms at orthologous nuclear gene loci among distant species in the conifer *Picea *(Pinaceae): Implications for the long-term maintenance of genetic diversity in trees. Am J Bot.

[B25] Pelgas B, Bousquet J, Beauseigle S, Isabel N (2005). A composite linkage map from two crosses for the species complex *Picea mariana *[Mill.] B.S.P x *Picea rubens *(Sarg.) and analysis of synteny with other Pinaceae. Theor Applied Genetics.

[B26] Neale DB, Savolainen O (2004). Association genetics of complex traits in conifers. Trends Plant Sci.

[B27] Gene Ontology Consortium (2001). Creating the gene ontology resource: design and implementation. Genome Res.

[B28] SpruceDB. http://ccgb.umn.edu/Pub_SpruceDB/.

[B29] Vettore AL, da Silva FR, Kemper EL, Souza GM, da Silva AM, Ferro MI, Henrique-Silva F, Giglioti EA, Lemos MV, Coutinho LL, Nobrega MP, Carrer H, Franca SC, Bacci Junior M, Goldman MH, Gomes SL, Nunes LR, Camargo LE, Siqueira WJ, Van Sluys MA, Thiemann OH, Kuramae EE, Santelli RV, Marino CL, Targon ML, Ferro JA, Silveira HC, Marini DC, Lemos EG, Monteiro-Vitorello CB, Tambor JH, Carraro DM, Roberto PG, Martins VG, Goldman GH, de Oliveira RC, Truffi D, Colombo CA, Rossi M, de Araujo PG, Sculaccio SA, Angella A, Lima MM, de Rosa Junior VE, Siviero F, Coscrato VE, Machado MA, Grivet L, Di Mauro SM, Nobrega FG, Menck CF, Braga MD, Telles GP, Cara FA, Pedrosa G, Meidanis J, Arruda P (2003). Analysis and functional annotation of an expressed sequence tag collection for tropical crop sugarcane. Genome Res.

[B30] Forment J, Gadea J, Huerta L, Abizanda L, Agusti J, Alamar S, Alos E, Andres F, Arribas R, Beltran JP, Berbel A, Blazquez MA, Brumos J, Canas LA, Cercos M, Colmenero-Flores JM, Conesa A, Estables B, Gandia M, Garcia-Martinez JL, Gimeno J, Gisbert A, Gomez G, Gonzalez-Candelas L, Granell A, Guerri J, Lafuente MT, Madueno F, Marcos JF, Marques MC, Martinez F, Martinez-Godoy MA, Miralles S, Moreno P, Navarro L, Pallas V, Perez-Amador MA, Perez-Valle J, Pons C, Rodrigo I, Rodriguez PL, Royo C, Serrano R, Soler G, Tadeo F, Talon M, Terol J, Trenor M, Vaello L, Vicente O, Vidal Ch, Zacarias L, Conejero V (2005). Development of a *citrus *genome-wide EST collection and cDNA microarray as resources for genomic studies. Plant Mol Biol.

[B31] Kawai J, Shinagawa A, Shibata K, Yoshino M, Itoh M, Ishii Y, Arakawa T, Hara A, Fukunishi Y, Konno H, Adachi J, Fukuda S, Aizawa K, Izawa M, Nishi K, Kiyosawa H, Kondo S, Yamanaka I, Saito T, Okazaki Y, Gojobori T, Bono H, Kasukawa T, Saito R, Kadota K, Matsuda H, Ashburner M, Batalov S, Casavant T, Fleischmann W, Gaasterland T, Gissi C, King B, Kochiwa H, Kuehl P, Lewis S, Matsuo Y, Nikaido I, Pesole G, Quackenbush J, Schriml LM, Staubli F, Suzuki R, Tomita M, Wagner L, Washio T, Sakai K, Okido T, Furuno M, Aono H, Baldarelli R, Barsh G, Blake J, Boffelli D, Bojunga N, Carninci P, de Bonaldo MF, Brownstein MJ, Bult C, Fletcher C, Fujita M, Gariboldi M, Gustincich S, Hill D, Hofmann M, Hume DA, Kamiya M, Lee NH, Lyons P, Marchionni L, Mashima J, Mazzarelli J, Mombaerts P, Nordone P, Ring B, Ringwald M, Rodriguez I, Sakamoto N, Sasaki H, Sato K, Schonbach C, Seya T, Shibata Y, Storch KF, Suzuki H, Toyo-oka K, Wang KH, Weitz C, Whittaker C, Wilming L, Wynshaw-Boris A, Yoshida K, Hasegawa Y, Kawaji H, Kohtsuki S, Hayashizaki Y, RIKEN Genome Exploration Research Group Phase II Team and the FANTOM Consortium (2001). Functional annotation of a full-length mouse cDNA collection. Nature.

[B32] Whitfield Ch.W, Band MR, Bonaldo MF, Kumar ChG, Liu L, Pardinas JR, Robertson HM, Soares MB, Robinson GE (2002). Annotated expressed sequence tags and cDNA microarrays for studies of brain and behavior in the honey bee. Genome Res.

[B33] Brown GR, Gill GP, Kuntz RJ, Langley CH, Neale DB (2004). Nucleotide diversity and linkage disequilibrium in loblolly pine. Proc Natl Acad Sci USA.

[B34] Pot D, McMillan L, Echt C, Le Provost G, Garnier-Géré P, Cato S, Plomion C (2005). Nucleotide variation in genes involved in wood formation in two pine species. New Phytologist.

[B35] Garcia-Gil MR, Mikkonen M, Savolainen O (2003). Nucleotide diversity at two phytochrome loci along a latitudinal cline in *Pinus sylvestris*. Mol Ecol.

[B36] Crow JA (2005). Diogenes – Reliable prediction of protein-encoding regions in short genomic sequences. http://analysis.ccgb.umn.edu/diogenes.

[B37] PFAM database. http://www.sanger.ac.uk/Software/Pfam/.

[B38] Bateman A, Coin L, Durbin R, Finn RD, Hollich V, Griffiths-Jones S, Khanna A, Marshall M, Moxon S, Sonnhammer EL, Studholme DJ, Yeats C, Eddy SR (2004). The Pfam Protein Families Database. Nucleic Acids Res.

[B39] Bairoch A, Apweiler R, Wu CH, Barker WC, Boeckmann B, Ferro S, Gasteiger E, Huang H, Lopez R, Magrane M, Martin MJ, Natale DA, O'Donovan C, Redaschi N, Yeh LL (2005). The Universal Protein Resource (UniProt). Nucleic Acids Res.

[B40] Rhee SY, Beavis W, Berardini TZ, Chen G, Dixon D, Doyle A, Garcia-Hernandez M, Huala E, Lander G, Montoya M, Miller N, Mueller LA, Mundodi S, Reiser L, Tacklind J, Weems DC, Wu Y, Xu I, Yoo D, Yoon J, Zhang P (2003). The *Arabidopsis *Information Resource (TAIR): a model organism database providing a centralized, curated gateway to *Arabidopsis *biology, research materials and community. Nucleic Acids Res.

[B41] Pine Gene Index PGI5.0 database. http://www.tigr.org/tigr-scripts/tgi/T_index.cgi?species=pine.

[B42] Davuluri RV, Sun H, Palaniswamy SK, Matthews N, Molina C, Kurtz M, Grotewold E (2003). AGRIS: *Arabidopsis *gene regulatory information server, an information resource of *Arabidopsis *cis-regulatory elements and transcription factors. BMC Bioinformatics.

[B43] Girke T, Lauricha J, Tran H, Keegstra K, Raikhel N (2004). The Cell Wall Navigator database. A systems-based approach to organism-unrestricted mining of protein families involved in cell wall metabolism. Plant Physiol.

[B44] Raes J, Rohde A, Christensen JH, Van de Peer Y, Boerjan W (2003). Genome-wide characterization of the lignification toolbox in *Arabidopsis*. Plant Physiol.

[B45] Li F, Zhang D, Fujise K (2001). Characterization of fortilin, a novel antiapoptotic protein. J Biol Chem.

[B46] Pay A, Heberle-Bors E, Hirt H (1992). An alfalfa cDNA encodes a protein with homology to translationally controlled human tumor protein. Plant Mol Biol.

[B47] Sage-Ono K, Ono M, Harada H, Kamada H (1998). Dark-induced accumulation of mRNA for a homolog of translationally controlled tumor protein (TCTP) in *Pharbitis*. Plant Cell Physiol.

[B48] Campbell M, Sederoff RR (1996). Variation in lignin content and composition. Plant Physiol.

[B49] Peleman J, Saito K, Cottyn B, Engler G, Seurinck J, Van Montagu M, Inze D (1989). Structure and expression analyses of the S-adenosylmethionine synthetase gene family in *Arabidopsis thaliana*. Gene.

[B50] Shen B, Li C, Tarczynski MC (2002). High free-methionine and decreased lignin content result from a mutation in the *Arabidopsis *S-adenosyl-l-methionine synthetase 3 gene. Plant J.

[B51] Sanchez-Aguayo I, Rodriguez-Galan JM, Garcia R, Torreblanca J, Pardo JM (2004). Salt stress enhances xylem development and expression of S-adenosyl-L-methionine synthase in lignifying tissues of tomato plants. Planta.

[B52] Espartero J, Pintor-Toro JA, Pardo JM (1994). Differential accumulation of S-adenosylmethionine synthetase transcripts in response to salt stress. Plant Mol Biol.

[B53] Schröder G, Eichel J, Breining S, Schröder J (1997). Three differentially expressed S-adenosylmethionine synthetase from *Catharantus roseus *: molecular and functional characterization. Plant Mol Biol.

[B54] Gramene database. http://www.gramene.org/.

[B55] Lindroth AM, Saarikoski P, Flygh G, Clapham D, Gronroos R, Thelander M, Ronne H, von Arnold S (2001). Two S-adenosylmethionine synthetase-encoding genes differentially expressed during adventitious root development in *Pinus contorta*. Plant Mol Biol.

[B56] CCGB biodata database. http://ccgb.umn.edu/biodata/.

[B57] Lamblin AF, Crow JA, Johnson JE, Silverstein KA, Kunau TM, Kilian A, Benz D, Stromvik M, Endre G, VandenBosch KA, Cook DR, Young ND, Retzel EF (2003). MtDB: a database for personalized data mining of the model legume *Medicago truncatula *transcriptome. Nucleic Acids Res.

[B58] Arborea project. http://www.arborea.ulaval.ca/en.

[B59] Chang S, Puryear J, Cairney J (1993). A simple and efficient method for isolating RNA from pine trees. Plant Mol Biol Rep.

[B60] Sambrook J, Fritsch EF, Maniatis T (1989). Molecular Cloning: A Laboratory Manual.

[B61] Yang GS, Stott JM, Smailus D, Barber SA, Balasundaram M, Marra MA, Holt RA (2005). High-throughput sequencing: a failure mode analysis. BMC Genomics.

[B62] Ewing B, Hillier L, Wendl MC, Green P (1998). Base-calling of automated sequencer traces using phred. I. Accuracy assessment. Genome Res.

[B63] Phrap software. http://www.phrap.org.

[B64] National Center for Biotechnology Information. http://www.ncbi.nlm.nih.gov/.

[B65] Altschul SF, Madden TL, Schäffer AA, Zhang J, Zhang Z, Miller W, Lipman DJ (1997). Gapped BLAST and PSI-BLAST: a new generation of protein database search programs. Nucleic Acids Res.

[B66] The Institute for Genomic Research. http://www.tigr.org/.

[B67] Brenner ED, Stevenson DW, McCombie RW, Katari MS, Rudd SA, Mayer KF, Palenchar PM, Runko SJ, Twigg RW, Dai G, Martienssen RA, Benfey PN, Coruzzi GM (2003). Expressed sequence tag analysis in *Cycas*, the most primitive living seed plant. Genome Biol.

[B68] Sputnik database. http://sputnik.btk.fi/.

[B69] The *Arabidopsis *Information Resource. http://www.arabidopsis.org.

[B70] Rice Genome Annotation Database. http://www.tigr.org/tdb/e2k1/osa1/.

[B71] EMBOSS. http://emboss.sourceforge.net/.

