# Supplementary material for: Generation, annotation, analysis and database integration of 16,500 white spruce EST clusters
Source: BMC Genomics. 2005 Oct 19;6:144. doi: 10.1186/1471-2164-6-144 (PMC1277824; doi:10.1186/1471-2164-6-144)
Supplement: Additional File 1 — Description of tissues used for cDNA library synthesis: genotype, treatments (type, level and duration), organ, tissue and developmental stage. [file 1471-2164-6-144-S1.doc]

**Additional file1: Description of tissues used for cDNA library synthesis: genotype, treatments (type, level and duration), organ, tissue and developmental stage**

| Library ID | Library Name | Genotype | Organ | Tissue | Stage |
| --- | --- | --- | --- | --- | --- |
| GQ001 | Male strobili development sequence | 13-272 | Expanding whole male strobili | Reproductive | Preformed male strobili at the end of winter dormancy; three stages were pooled: swollen fully closed buds, partly open buds and fully open buds |
| GQ002 | Female cones development sequence | 13-272 | Expanding whole female cones | Reproductive | Preformed female cones at the end of winter dormancy; three stages were pooled: swollen fully closed buds, partly open buds and fully open buds |
| GQ003 | Vegetative buds development sequence | 13-272 | Expanding whole vegetative buds | Needles and primary shoot | Preformed vegetative buds at the end of winter dormancy; three stages were pooled: swollen fully closed buds, partly open buds and fully open buds |
| GQ004 | Secondary xylem -mature trees | Two trees of provenance 5333 and one from 5206 | Stem from ground to lower part of live crown, on 33 year old tree | Non-lignified differentiating secondary xylem from normal vertical trees during formation early wood | Non-lignified xylem from trees harvested 2.5 hours, 6 hours and 11 hours after day break |
| GQ005 | Partly-lignified secondary xylem from mature trees | Two trees of provenance 5333 and one from 5207 | Stem from ground to lower part of live crown, on 33 year old tree | Partly-lignified differenting secondary xylem from normal verticaltrees during formation early wood | Partly-lignified xylem from trees harvest 2.5 hours, 6 hours and 11 hours after day break |
| GQ006 | Cambium-Phloem - mature trees | Two trees of provenance 5333 and one from 5208 | Stem from ground to lower part of live crown, on 33 year old tree | Cambium and phloem region from normal vertical trees during formation early wood | Cambium and phloem tissue scrapped from inside of bark from trees harvest 2.5 hours, 6 hours and 11 hours after day break |
| GQ007 | Secondary xylem - guirdled seedlings | pg-653 | Main stem region producing secondary growth on 60 cm tall seedlings | Secondary xylem tissues of girdled plants, above and below girdling point. Pooled tissues collected 1 and 7 days after guirdling treatment on actively growing seedlings | Xylem at all stages of differentiation and wood |
| GQ008 | Cambium, Phloem, Bark -guirdled seedlings | pg-653 | Main stem region producing secondary growth on 60 cm tall seedlings | Cambium, phloem,and bark of girdled plants, above and below girdling point | Pooled tissues collected 1 and 7 days after guirdling treatment on actively growing seedlings |
| GQ013 | Elongating roots tips - sapplings | pg-653 | Roots from 60 cm tall seedlings. | Tender roots tips free of michorizee | Actively elongating roots |
| GQ016 | Primary, secondary shoot -N fertilization Treatments | pg-653 | Main stem region producing secondary growth on 60 cm tall seedlings | Xylem, pith, cambium, phloem, bark | Primary & secondary shoot, secondary phloem pooled from plants fertilized with low and high NH4NO3 |
| GQ017 | Immature somatic embryos | pg-653 | Whole intact somatic embryos comprised comprised of senescing suspensor and bullet stage embryonic head | Whole embryo | Collected after two-weeks on maturation medium containing ABA |
| GQ018 | Clean roots systems - N treatments | pg-653 | whole root systems form 15 cm tall seedlings | Whole roots systems, predominantly free of michorizee | Roots from plants fertilized with low and high NH4NO4 |
| GQ019 | Clean roots systems - P treatments | pg-653 | whole root systems form 15 cm tall, 4 month old seedlings produced by somatic embryogenesis | Whole roots systems, predominantly free of michorizee | Tissues were pooled from several plants receiving daily fertilization varying potasium levels (either 0.02 mM or 2 mM PO4), collected at 4 hours, 24 hours, 2 days, 4 days or 7 days of treatment |
| GQ020 | Clean roots systems -Diurnal cycle | pg-653 | whole root systems form 15 cm tall, 4 month old seedlings produced by somatic embryogenesis | Whole roots systems, predominantly free of michorizee | Tissues were pooled from several plants grown under 20h light/4h dark and collected every 2 hours over a period of 28 hours (4 dark time points and 11 time points in light). |
| GQ022 | Root xylem - mature trees | Three trees Unknown | Roots from 9 year old trees measuring approximately 4.5 m tall, and 10 cm in diameter. | Differentiating xylem from roots 1 cm in diameter or larger | Non-lignified secondary xylem tissues from large diameter roots in early part of growing season (June). |
| GQ025 | Annual flush shoots diurnal cylce- trees | Several trees Unknown | Stem annual flush from top of 10 year old trees, approximately 5 m tall and 12 cm in diameter | Pith, xylem, cambium, phloem and bark. Needles were removed. | Shoot comprised of primary and secondary growth (no needles) of annual flush pooled from several trees collected over a natural 15h40min light / 9h20min dark cycle every three hours |
| GQ026 | Needles - N fertilization treatments | pg-653 | Foliage stem from 60 cm tall seedlings | Needles from plant fertilized with 0 mM NH4NO3 (low N) AND with 10 mM NH4NO3 (high N) | Young and mature needles were pooled |
