# Supplementary material for: Generation, annotation, analysis and database integration of 16,500 white spruce EST clusters
Source: BMC Genomics. 2005 Oct 19;6:144. doi: 10.1186/1471-2164-6-144 (PMC1277824; doi:10.1186/1471-2164-6-144)
Supplement: Additional File 2 — Annotation of proteins related to the cell wall based on similarities with sequences from the Cell Wall Navigator Database [44]and lignin biosynthesis enzymes [45]. Spruce homologs were identified by tblastx searches with e-value < 1e-10. [file 1471-2164-6-144-S2.doc]

**Additional file 2: Annotation of proteins related to the cell wall based on similarities with sequences from the Cell Wall Navigator Database [44] and lignin biosynthesis enzymes [45].** Spruce homologs were identified by *tblastx* searches with e-value<1e-10.

| A-Cell wall related proteins described in the Cell Wall Navigator DB | | | Number of contigs |
| --- | --- | --- | --- |
| 1 Monosaccharide activation and interconversion | | | |
|  | 1.1 Sugar 1-kinases (S1K) | | 2 |
|  | 1.2 Nucleotide-sugar pyrophosphorylases | |  |
|  |  | 1.2.1 GDP-mannose pyrophosphorylase (GMP) | 11 |
|  |  | 1.2.2 UDP-glucose pyrophosphorylases (UGP) | 5 |
|  | 1.3 Nucleotide-sugar interconversion enzymes | |  |
|  |  | 1.3.1 Nucleotide-sugar interconversion enzymes | 50 |
|  |  | 1.3.2 Nucleotide sugar dehydrogenase superfamily (UGD) | 7 |
|  |  |  |  |
| 2 Polysaccharide synthesis | | | |
|  | 2.1 Cellulose and Galactomannan | |  |
|  |  | 2.1.1 Cellulose and beta-mannan synthase-like (CSL) | 29 |
|  | 2.2 Hemicellulose | |  |
|  |  | 2.2.1 Reversibly glycosylated polypeptides (RGP) | 2 |
|  |  | 2.2.2 Xyloglucan galactosyltransferases (XGT) | 34 |
|  |  | 2.2.3 Xyloglucan fucosyltransferases (XFT) | 0 |
|  |  | 2.2.4 Xyloglucan xylosyltransferases and galactomannan galactosyltransferases (XXT) | 3 |
|  | 2.3 Callose | |  |
|  |  | 2.3.1 Glucan synthase-like (GSL) | 5 |
|  | 2.4 Other glycosyl transferases | |  |
|  |  | 2.4.1 Glycosyl transferases (pectin bios.) (GT8) | 35 |
|  |  |  |  |
| 3 Reassembly | | | |
|  | 3.1 Cell Expansion | |  |
|  |  | 3.1.1 Expansins (EXP) | 30 |
|  |  | 3.1.2 Yieldins (GH18) | 18 |
|  | 3.2 Hemicellulose reassembly | |  |
|  |  | 3.2.1 Xyloglucan endotransglycosylases/hydrolases (XTH) | 56 |
|  | 3.3 Glycoside hydrolases | |  |
|  |  | 3.3.1 Beta-galactosidases (BGAL) | 0 |
|  |  | 3.3.2 Glycoside hydrolases 9 (GH9) | 15 |
|  |  | 3.3.3 Glycoside hydrolases 17 (GH17) | 3 |
|  |  | 3.3.4 Polygalacturonases (GH28) | 22 |
|  |  | 3.3.5 Glycoside Hydrolase Family 10 (GH10) | 3 |
|  |  | 3.3.6 Glycoside Hydrolase Family 43 (GH43) | 7 |
|  |  | 3.3.7 Alpha-L-arabinofuranosidases (GH51) | 4 |
|  | 3.4 Lyases | |  |
|  |  | 3.4.1 Pectate and pectin lyases (PL1) | 19 |
|  |  | 3.4.2 Rhamnogalacturonan I lyases (PL4) | 7 |
|  | 3.5 Esterases | |  |
|  |  | 3.5.1 Pectin methyl esterases (PME) | 50 |
|  |  | 3.5.2 Pectin acetylesterases (PAE) | 8 |
|  |  | 3.5.3 Feruloyl esterases (FE) | 1 |
|  |  |  |  |
| 4 Structural proteins | | | |
|  | 4.1 Hydroxyproline-rich glycoproteins (HRGP) | | 60 |
|  | 4.2 Leucine-rich repeat extensins (LRX) | | 180 |
|  | 4.3 Proline-rich proteins (PRP) | | 0 |
|  | 4.4 Glycine-rich proteins (GRP) | | 0 |
|  | 4.5 Arabinogalactan proteins (AGP) | | 22 |
|  |  |  |  |
| 5 Glycoprotein glycosyltransferases | | | |
|  | 5.1 Glycoprotein fucosyltransferases (GFT) | | 2 |
|  | 5.2 Glycosyl transferases 21A (GT31a) | | 11 |
|  | 5.3 Glycosyl transferases 31B (GT31b) | | 7 |
| Total (Cell Wall Navigator comparison) | | | 708 |

B- Lignin biosynthesis enzymes

|  | 4-coumarate: coenzyme A ligase (4CL) | 4 |
| --- | --- | --- |
|  | Coumarate 3-hydroxylase (C3H) | 6 |
|  | Cinnamate 4-hydroxylase (C4H) | 1 |
|  | Cinnamoyl alcohol dehydrogenase (CAD) | 10 |
|  | Caffeoyl-CoA O-methyltransferase (CCoAOMT) | 4 |
|  | Cinnamoyl CoA reductase (CCR) | 5 |
|  | Caffeic acid O-methyltransferase (COMT) | 12 |
|  | Hydroxycinnamoyltransferase (HCT) | 4 |
|  | Phenylalanine ammonia lyase (PAL) | 1 |
| Total (lignin) | | 47 |
